# Effect of D-003, a Mixture of High Molecular Weight Aliphatic Acids, on Glucocorticoid- and Lipopolysaccharides (LPS)-Induced Osteonecrosis 

**Published:** 2012

**Authors:** Miriam Noa, Rosa Más, Maikel Valle, Sarahí Mendoza, Nilda Mendoza

**Affiliations:** *Center of Natural Products from the National Center for Scientific Research, Havana City, Cuba. *

**Keywords:** D-003, Osteonecrosis, Glucocorticoid, Lipopolysaccharides

## Abstract

Osteonecrosis (ON) is characterized through the impairment of osseous blood flow that leads to the collapse of femur head. Corticoid-induced ON in rats and lipopolysaccharide (LPS)-induced in rabbits are useful models to assess the efficacy of potential treatments on this disease**. **D-003 inhibits the mevalonate pathway, lipid peroxidation and prevents osteoporosis in rats through increasing the osteoclast apoptosis. This study investigated the effects of D-003 on corticoid- and LPS-induced ON in rats and rabbits.

*Corticoid-induced ON*: Rats were randomized into five groups. A negative control and four groups treated with prednisolone 6 mg/Kg: a positive control and three treated with D-003 (5, 25 and 200 mg/Kg) for 80 days. All positive controls presented ON areas. D-003 significantly reduced the numbers and proportions of ON lesions, as compared to the positive control group.

*LPS-induced ON in rabbits*: Rabbits were randomized into five groups: a negative control and four injected with a single intra-venous injection of LPS (10 μg/Kg) including a positive control and three with D-003 (5, 25 and 200 mg/Kg) for 30 days. ON was seen in all positive controls. The incidence of ON and the number of ON lesions in the groups treated with D-003 (25 and 200 mg/Kg) was significantly lower compared to the positive controls. LPS injection significantly increased the size of bone marrow fat cells in positive controls and such increase was significantly decreased by D-003. In conclusion, D-003 reduced ON lesions in corticoid-and LPS-induced ON and also the size of bone marrow fat cells in rabbits with LPS.

## Introduction

Osteonecrosis (ON) is a bone disease characterized through the impairment of osseous blood flow that lead to the collapse of femur head, eventually requiring hip arthroplasty which may consequently impair the quality of sufferers’ life (1). 

ON may be post-traumatic or non-traumatic and results from a multifactorial process that depends of both genetic predisposition and exposure to risk factors. Animal models of ON that partially mimic the features of human’s ON, have been useful to study the mechanisms whereby ON is developed and to assess the efficacy of potential treatments on this disease. Among them, models of spontaneous, surgically-induced (traumatic) and non-traumatic ON have been developed. Since the long-term use of corticosteroids (drugs mainly aimed at treating the inflammatory diseases) is a major risk factor for developing non-traumatic ON (1, 2), the models of corticoids-induced ON have been widely used (2-4). Besides, a model of lipopolysaccharide (LPS)-induced non-traumatic ON in rabbits has been useful to assess the pathogenesis and to investigate the putative value of some treatments on the corticoid-independent non-traumatic ON in humans (5-8).

Although the pathogenesis of ON is not fully understood, different theories explain the underlying mechanisms of steroid-induced ON, like the increased size and number of bone marrow fat cells, increased intra-osseous pressure, fatty degeneration of osteocytes, fat embolism and extraosseous arterial occlusion, coagulation abnormalities and hyperlipidaemia (5-9). Experimental studies suggest that alcohol-induced adipogenesis of bone marrow stromal cells may lead to the ON of the femoral head. (8)

Nowadays, no treatment is actually effective to prevent or treat ON, but bisphosphonates, lipid-lowering drugs, anticoagulants and vasodilators are used to manage this disease since they may reduce the risk factors for ON. Since abnormal lipid metabolism and coagulopathy have been linked with ON (10), lipid-lowering agents (11-13), anticoagulants and prostacyclin analogues (14, 15) have been used to treat ON, whereas the use of bisphosphonates is based on the reduction of osteoclasts activity (16-18).

D-003 is a reproducible mixture of very long-chain aliphatic acids purified from sugarcane wax, wherein octacosanoic, triacontanoic, dotriacontanoic, and tetratriacontanoic acids are the most abundant (19). D-003 inhibits cholesterol synthesis prior to mevalonate formation through regulating HMG-CoA reductase activity (20) and displays the cholesterol-lowering effects with reduction of low density lipoprotein (LDL-C), and increase of the high density lipoprotein (HDL-C) (21-24) that has also been shown to inhibit the lipid peroxidation in experimental (25) and clinical (23) studies.

Consistently with the inhibitory effect of D-003 on the mevalonate to cholesterol pathway (20), oral-therapy with D-003 (5-200 mg/Kg) has been shown to prevent bone loss and bone resorption in ovariectomized (ovx) rats (26-29) through increasing osteoclast apoptosis (26, 27) and in prednisolone-induced osteoporosis in rats (30).

In light of this background, this study investigated the *in-vivo *effects of D-003 on corticoid- and LPS-induced ON in rats and rabbits, respectively.

## Experimental

*Animals*

Female Sprague Dawley rats (225 ± 20 g) aged 3 months and adult (defined as having the growth plate already closed) male F_1 _rabbits (3-3.5 Kg), 30-34 weeks old, from the National Center for Laboratory Animals Production (CENPALAB, Havana, Cuba) were adapted to laboratory conditions temperature of 21°C, humidity of 55%, 12 h light/dark cycles) for 2 weeks with free access to food (rodent and rabbit chow from CENPALAB, respectively) and water.

An independent board approved the use of the animals in the experiment and animal handle was conducted according to the Cuban regulations for the use of laboratory animals. Study conduction was consistent with the approved protocol.

*Administration and dosage*

The batch of D-003 used in the experiment was obtained from the Chemistry Department of the Center of Natural Products (Havana City, Cuba). The composition of this batch, assessed through a validated gas chromatography method (31), was as follows (w/w): 1-tetracosanoic (0.3%), 1-pentacosanoic (0.5%), 1-hexacosanoic (2.4%), 1-heptacosanoic (2.6%), 1-octacosanoic (37.1%), 1-nonacosanoic (1.8%), 1-triacontanoic (17.7%), 1-hentriacontanoic (1.3%), 1-dotriacontanoic (8.9%), 1-tritriacontanoic (1.1%), 1-tetratriacontanoic (10.9%), 1-pentatriacontanoic (0.6%) and 1-hexatriacontanoic (3.8%) acids. Batch purity (total content of these acids) was 89.1%.

For dosing, D-003 was suspended in a 2% Tween 20/water vehicle. After corroborating their stability in the vehicle, suspensions were prepared weekly by adjusting their concentrations to the animal bodyweight gain.

Corticoid-induced ON in rats was induced through administering orally prednisolone 6 mg/Kg for 80 days as described by Oxtaf and Oxlund (32). Rats were randomized into five groups of 10 as follows: a negative control, treated orally with the vehicle, and four prednisolone-treated groups, a positive control orally treated with the vehicle and three treated with D-003 (5, 25 and 200 mg/Kg, respectively). Treatments were administered once a day from 9 to 11 a.m. for 80 days.

The LPS used for inducing ON, were gently donated by the Vaccine Department of the Biotechnology Branch of the National Center for Scientific Research (Havana City, Cuba), was isolated from a strain of *Vibrio cholerae *serology 01, biotype El Tor, serotype Ogawa and purified by gel filtration. LPS was reconstituted through adding 1 mL of sterile balanced salt solution.

For the induction of ON, rabbits were administered intra-venous (IV) injection of LPS (10 μg/Kg), according to Irisa *et al. *(2001) (5). Rabbits were randomized into five groups: a negative control not injected with LPS and treated orally with the vehicle (6 rabbits), and four LPS-injected groups (8 rabbits per group): one orally treated with the vehicle (positive control) and three with D-003 (5, 25 and 200 mg/Kg, respectively). Treatments were given once a day from 9 to 11 a.m. for 30 days.

The lowest dose of D-003 used (5 mg/Kg) has been shown to lower cholesterol in rabbits (22), to inhibit lipid peroxidation in rats (26) and to prevent bone loss and bone resorption in ovx rats (27-30), and in rats with corticoid-induced osteoporosis (30).

*Bodyweight control*

In both experiments, body weights were controlled at baseline (the day before starting the treatments) and every 15 days thereafter.

*Microscopic studies*

*Corticoid-induced ON in rats*

At study completion, rats were fasted for 12 h and sacrificed through exsanguination under ether anaesthesia. Then, the right femur and fifth lumbar vertebrae from each rat were removed for the morphological study.

The study was conducted as described previously (30, 32). Bone samples (eight regions per rat) were examined under a light microscope for histopathological changes, like hematopoietic cell necrosis with cytolysis, karyorrhexis and/or karyolysis, fat cell necrosis with the loss of nuclei and distinct cell borders. ON was blindly diagnosed. The occurrence of ON was considered if bone necrosis showing empty lacunae or pycnotic nuclei of osteocytes and surrounding bone marrow necrosis (viz, necrosis of adipocytes and hematopoietic cells) were present. All rats that had at least one osteonecrotic lesion out of eight areas were considered to have ON, whereas those with no osteonecrotic lesions were considered ON-free.

*LPS-induced ON in rabbits*

At study completion, rabbits were fasted for 12 h, anaesthetized with sodium pentobarbital (30 mg/Kg IV) and then killed through exsanguination via aortectomy.

Treatment effects were assessed through microscopic and morphometric studies. In this model, ON is reported to be present bilaterally in almost all rabbits (5). Then, we removed the right femur of each animal for the morphological study.

Bones were decalcified in 0.5 M disodium ethylenediaminetetraacetic acid (EDTA, pH = 7.4) at 4°C for 4 weeks, embedded in paraffin, sectioned and stained with haematoxylin and eosin (33). The bone samples were cut along the coronal plane in the proximal one-third and axial plane in the distal part (condyle).

The diagnosis of ON was determined 4 weeks after the LPS administration, since at this time development, ON has been documented (5). Whole areas of the proximal one-third and distal condyles of the femur (totaling 8 regions) were examined histopathologically. The frequency and location of ON and numbers of ON lesions per group were measured.

ON was blindly diagnosed. A positive diagnosis was based on the diffuse presence of empty lacunae or pyknotic nuclei of osteocytes within the bone trabeculae, accompanied by surrounding bone marrow cell necrosis (cytolysis, karyorrhexis and/or karyolysis, fat cell necrosis with the loss of nuclei and distinct cell borders) (5). Rabbits with at least 1 osteonecrotic lesion in the 8 areas examined were considered to have ON, whereas those without osteonecrotic lesions were classified as ON-free.

We determined both the osteonecrotic lesions per rabbit (maximum 8 regions) and the numbers of rabbits with ON (5). Necrotic areas of rabbits with ON were morphometrically measured in the proximal one-third of the femur on the coronal sections at the maximal width. Necrotic rate was calculated as a percentage of necrotic area per the total area examined (5).

We determined the size of bone marrow fat cells with clearly defined profile in 4 randomly selected fields (up-down-left-right) of each dissected part of proximal one-thirds and distal condyles of both femora and humerus (32 fields for 8 dissected parts from each rabbit), as per the criteria of Motomura *et al. *(11). Fat cells that had undergone necrosis were excluded from this study. The repeatability of this measurement method has been confirmed in previous study (14).

*Statistics*

Data were expressed as mean ± SD and percentages. Continuous data (bodyweights, numbers of ON lesions on a determined bone volume area, area percentage of ON, and sizes of bone marrow fat cells) were compared with the non-parametric Mann-Whitney U-test. Categorical data (proportions of ON-positive animals) were compared with the Fisher’s Exact Probability Test. A α = 0.05 was *a priori *selected for statistical significance. Statistical analyses were performed using the software Statistics for Windows (Kernel release 5.1, Statsoft, Inc.1998, Tulsa, OK, USA).

## Results and Discussion

No significant changes on bodyweight gain of D-003-treated and control groups were found (data not shown for simplicity). In the experiment of LPS-induced ON, two control rabbits died within the first 24 h after the LPS injection.

*Corticoid-induced ON in rats*

All positive controls (100%) and none-negative control (0%) presented ON areas (Table 1.). 

**Table 1:** Effects of D-003 on prednisolone (PRED)-induced osteonecrosis (ON) in rats

**Group**	**Doses (mg/Kg)**	**Numbers of ON lesions ** **(X ± SD)**	**Inhibition%**	**Rats with ON/n (%)** **a**	**Inhibition%**
Negative control (Oral vehicle)	0	0***	-	0/10 +++ (0.0%)	-
Positive control (PRED + vehicle)	0	3.87 ± 1.46	-	10/10 (100%)	-
PRED + D-003	5	2.12 ± 2.95	45.2	3/10 + (30.0%)	70
PRED + D-003	25	1.37 ± 2.56*	64.5	2/10 ++ (20.0%)	80
PRED + D-003	200	0.25 ± 0.71***	93.5	1/10 +++ (10.0%)	90

X: mean, SD: standard deviations, a: number of lesions per rat/number of rats per group, * p < 0.001, Comparison with positive control, (Mann-Whitney U-test), + p < 0.05, ++ p < 0.01, +++ p < 0.001, Comparisons with positive control, (Fishers’ Exact Probability test)

D-003 (5, 25 and 200 mg/Kg) significantly reduced the numbers of ON lesions by 45.2%, 64.5% and 93.5%, respectively, and the proportions of rats with prednisolone-induced ON by 70, 80 and 90%, respectively, as compared to the positive control group. Furthermore, D-003 also practically abolished the prednisolone-induced occurrence of hypertrophy of bone marrow adipocytes and lipid-laden pluripotential stromal cells in the femoral bone.

*LPS-induced ON in rabbits*

Occurrence of ON of their femoral bone was seen in all positive control rabbits (8/8, 100%), mainly in the metaphyses and diaphyses and in lesser degree in femoral epiphyses and condyles. The incidence of ON in the groups treated with D-003 25 (3/8, 37.5%) and 200 mg/Kg (2/8; 25%) was significantly lower (p < 0.05 for both doses) than in the positive controls (6/6; 100%), so that D-003 (5, 25 and 200 mg/Kg), lowered ON rates by 62.5%, 75% and 88.5%, respectively, as compared to positive control rats. The reduction of ON was enhanced with the doses. Also, D-003 (5, 25 and 200 mg/Kg) significantly (p < 0.05 for all doses) and dose-dependently decreased the numbers of ON lesions by 45.2%, 64.5% and 93.5%, as compared to the positive control group (Table 2.).

**Table 2:** Effects of D-003 on lipopolysaccharides (LPS)-induced osteonecrosis (ON) in rabbits

**Group**	**Doses (mg/Kg)**	**Numbers of ON lesions ** **(X ± SD)**	**Inhibition%**	**Rabbits with ON n(%)**	**Inhibition%**
Negative control (Oral vehicle)	0	0***	-	0/6 + (0.0%)	-
Positive control (LPS + vehicle)	0	3.00 ± 1.67	-	8/8* (100%)	-
LPS + D-003	5	0.62 ± 0.92*	45.2	3/8 + (37.5%)	62.5
LPS + D-003	25	0.37 ± 0.74*	64.5%	2/8 ++ (25.0%)	75
LPS + D-003	200	0.14 ± 0.38*	93.5%	1/8 +++ (12.5%)	88.5

X mean, SD standard deviation, a number of lesions per rabbit/number of rabbits per group, * p < 0.05, ** p < 0.01, Comparisons with positive controls, (Mann Whitney U-test), + p < 0.05, ++ p < 0.01, +++ p < 0.001, Comparisons with positive control, (Fishers’ Exact Probability test

The average size of bone marrow fat cells significantly (p < 0.01) increased in the positive controls as compared to the negative control group, and such increase was significantly (p < 0.01) and markedly decreased by D-003 by 94.4% (5 mg/Kg) and 100% (25 and 200 mg/Kg) (Table 3.).

**Table 3:** Effect of D-003 on the size of bone marrow fat cell sizes LPS-induced ON in rabbits

**Groups**	**D-003 oral doses**	**Size of bone marrow fat cells (μm)**	**%I**
Negative control	0	55.48 ± 0.21	
Positive control	0	61.33 ± 0.38**	
D-003 + LPS	5	57.95 ± 0.33*	94.4
D-003 + LPS	25	56.31 ± 0.36 +++	100
D-003 + LPS	200	55.07 ± 0.33 +++	100

LPS lipopolysaccharides, ON osteonecrosis, X mean, SD standard deviation * p < 0.01 Comparisons with positive controls (Mann-Whitney U-test

The results here presented show that D-003 (5, 25 and 200 mg/Kg/day) prevented corticoid and LPS-induced ON in rats and rabbits, respectively.

As expected, prednisolone administration produced femoral ON with the presence of hypertrophy of bone marrow adipocytes and lipid-laden pluripotential stromal cells in the bones, which was evident in all positive control rats. These results are consistent with the report of other authors (34) who found that the bone marrow of dexamethasone-treated mice was infiltrated through numerous lipid-laden, pluripotential stromal cells (34, 35) and confer the validity to the data obtained in our experimental conditions, so that the absence of these prednisolone-induced findings into the femoral bone observed in D-003-treated groups described here can be attributable to D-003 treatment. Indeed, the effects of D-003 on prednisolone-induced ON were actually remarkable since the highest dose (200 mg/Kg) decreased the numbers of ON lesions by 93.5% and the rate of rats with ON by 90% as compared to the positive controls. The lowest dose (5 mg/Kg) also produced meaningful reductions related to the number of ON lesions (45.2%) and animals with ON (70%).

The great reduction of hypertrophic bone marrow adipocytes in D-003-treated rats could explain the reduction of prednisolone-induced ON observed in our study, since the lipid-induced hypertrophy of the fat cells of rats treated with glucocorticoids cannot expand the marrow cavity within the inflexible osseous cage, which in turn leads to increased intra-osseous pressure, sinusoidal compression, venous stasis and eventual arterial obstruction, producing ischemic ON (34).

LPS-induced mortality findings are consistent with previous reports (5). Rabbits died mainly due to the circulatory disturbances; the overall mortality rate (2/38, 5.3%) was lower than that reported (11%) by other authors (5), but the mortality rates among positive controls (2/8, 25%) was greater than that described (5).

The effects of D-003 on LPS-induced ON matched well with its effects on glucocorticoid-induced ON. The results seen in our positive controls, all of which showed multifocal and widespread ON lesions, are consistent with those expected, which supports the reproduction of this model in our conditions. The preventive effects of D-003 (5-200 mg/Kg) on LPS-induced ON were of appreciable magnitude, as it decreased the ON rates from 62.5% to 88.5%, and the numbers of ON lesions from 45.2% to 93.5%, as compared to the positive controls.

LPS-induced ON emerges as the consequence of several factors since LPS is a constituent of the cell wall of Gram-negative bacilli that exhibits several pharmacological effects, like immune activation, induction of inflammation-circulatory disturbances, so that it may trigger intra-vascular coagulation, fat embolism and hyperlipidemia, among others. It is relevant to note that LPS activates the vascular endothelial cells, platelets, monocytes/macrophages and components that lead to hypercoagulable and/or hypofibrinolytic states (5).

Although the increases in the size of bone marrow fat cells of rabbits injected with LPS had not been described before (Entrez PubMed reviewed up to May 2011), such finding is consistent with that referred to steroid (11) and alcohol-induced ON (8).

Although the elucidation of the mechanisms, whereby D-003 prevent non-traumatic corticoid and LPS-induced ON, are beyond the objective of this study, these effects could be related, at least partially, with some of the pharmacological actions of D-003, like its cholesterol-lowering (20) and antioxidant (23, 25) effects.

First, lipid-lowering treatments have been shown to reduce the ON occurrence though; the reduction of lipid deposition in bone marrow tissues is due to the decrease in size of bone marrow fat cells (11-13). Accordingly, the inhibitory effect of D-003 on the ON development may be associated with the reduction of lipid deposition in bone marrow tissues, as manifested through the reduction of hypertrophic bone marrow adipocytes in D-003-treated animals. These results are consistent with the effects of D-003 on corticoid-induced ON.

Second, the efficacy of D-003 in preventing LPS-induced ON can be also related to its antioxidant effects (23, 25), since *in-vivo *oxidative stress has been implicated in the development of steroid-induced ON in rabbits (35, 36) and rats (37). While glutathione depletion induces ON in rats (37) and steroids treatment induces ON in the rabbit (35, 36), treatment with glutathione decreases the incidence of ON (36). Lipid peroxidation may cause cytomembrane injury that induce the degeneration of arterioles and arteriolosclerosis, which eventually leads to ischemia in the target organ, including the femoral head. Furthermore, the direct cytotoxicity of lipid peroxidation products might damage ischemic osteocytes, leading to irreversible cell injury, death and ON (8).

Platelet activation and hypercoagulation states have been linked with the development of ON (14, 15, 38) so that the antiplatelet and anticoagulant agents should be useful to manage ON since they may enhance blood flow to ischemic bone areas and potentially promote the revascularization (14, 15). In such regard, the antiplatelet effects reported for D-003 (39, 40) could have contributed to the present results.

Finally, since bisphosphonates have been able to reduce ON due to the reduction of bone resorption (18), we cannot discard that the antiresorptive effects of D-003 (26-30) have been contributed partially to the prevention of corticoid and LPS-induced ON shown here.

The present results merits further studies on the potential benefits of D-003 for managing ON, a difficult-to-treat disease (2), but extensive experimental and clinical research is required to demonstrate whether D-003 can be actually useful in the ON management process.

## Conclusions

This study demonstrates that D-003 (5-200 mg/Kg) administered orally for 80 and 30 days reduced the incidence and numbers of ON lesions in corticoid- and LPS-induced ON, respectively, and the size of bone marrow fat cells in rabbits with LPS-induced ON. 
